# Orthopaedic health status of horses from 8 riding schools - a pilot study

**DOI:** 10.1186/1751-0147-52-50

**Published:** 2010-08-20

**Authors:** Agneta Egenvall, Cecilia Lönnell, Christopher Johnston, Lars Roepstorff

**Affiliations:** 1 Department of Clinical Sciences, Swedish University of Agricultural Sciences, Box 7054, SE-750 07 Uppsala, Sweden; 2 University Animal Hospital, Box 7018, SE-750 07 Uppsala, Sweden; 3 Department of Anatomy, Physiology and Biochemistry, Unit of Equine Studies, Swedish University of Agricultural Sciences, Box 7046, SE-750 07 Uppsala, Sweden

## Abstract

**Background:**

Orthopaedic injury is the most common reason for lameness and wastage in sport and leisure horses. Studies on racehorses have shown differences in injury risk between trainers and training strategies. The aim was to study between riding school variation in orthopaedic health status by clinical examination and horses age, and control for change of examiner, in schools with previous high (n = 4) and low (n = 4) insurance utilisation.

**Methods:**

Horses (n = 99) at 8 riding schools were examined for conformation, movement in all gaits, standing flexion tests and palpation by two veterinary surgeons (in some schools only one). Indexes of findings were created for total health, movements, limbs, conformation and back palpation.

**Results:**

Logistic regression analyses showed that findings increased with age (walk, trot, canter, conformation left hind limb, palpation fore limbs, hooves and flexion tests) or decreased with age (conformation right fore limb). Significant differences in findings were found between riding schools and examiner for seven and eight criteria each (partly overlapping). Increasing indexes were significantly associated with one examiner (total health, movements, back palpation), increasing age (total health, movements) or more time at the school (limbs). The back palpation index was highest at 5 < 8 years since acquisition.

**Conclusion:**

The age distribution differed markedly between riding schools and age affected several types of findings. This, combined with the two opposite groups of insurance use, shows that schools with low insurance utilisation had previously been able to "avoid" using the insurance, maybe even on similar types of cases if these were more promptly/differently handled indicating differential coverage of disease data in the insurance database. The examiner effect was clearly demonstrated. For some findings, the amount of clinical observations differed by school, even when examiner and age was adjusted for. Most findings were of minor importance, including slight movement irregularities. Orthopaedic status varies between riding schools. We hypothesize that this is associated with management factors that warrant further study.

## Introduction

Lameness is the most common problem in equine veterinary practice [1-3]. Studies of musculoskeletal injuries in Thoroughbred race horses have shown that the risk of injury is not equally distributed across the population, but varies with trainer and/or training regimens [4-9].

Riding schools represent an important proportion of the horse industry, not least in Sweden, with students taking ~eight million lessons annually [10]. An equine insurance database [11] has been used to study disease patterns in Swedish riding school horses [12]. Locomotor problems accounted for 70% of insurance claims in riding school horses. Between-school variation in utilisation of insurance was substantial. We hypothesised that differences in risk of clinical orthopaedic health problems, as shown by insurance utilization data, correspond to differences in orthopaedic health problems that can be detected by an experienced equine practitioner.

The aim was to study between riding school variation in orthopaedic health status and horses age, by clinical examination in riding schools with previous high and low insurance utilisation. Age/time variables, movement, conformation, clinical and orthopaedic status of horses, judged by experienced equine clinicians, were compared. Because the appointed main clinical examiner was not able to finish the study, emphasis has been put on controlling for the effect of change of examiner.

## Materials and methods

### Study population

Riding schools with an average of ≥8 horses insured annually 1997- 2002 for both life and veterinary care at Agria were selected, resulting in a list of 73 riding schools ranked relative to veterinary care and life insurance claims. From these, 4 high (HIU) and 4 low insurance utilisation (LIU) riding schools were selected, based on combined high and low previous utilisation of veterinary care and/or life insurance, geographical convenience and agreement to offer the horses for examination (two riding schools in the "first-selected" LIU group declined examination). The HIU schools had ranks for veterinary care insurance claims of 5, 2, 19 and 8 (a low rank equals a high rate of insurance claims). The HIU schools had ranks for life insurance claims of 2, 1, 3, and 5 (combined in order with the veterinary care insurance). The LIU schools had ranks for veterinary care of 71, 70, 65 and 37 and for life insurance the ranks were 71, 14, 69 and 72.

The study excluded ponies, as they tend to be a separate part of the riding schools with lighter work involving more children and beginners. All horses deemed to be fit for usage in riding lessons at time of the visit were examined. Lame or convalescent horses were excluded, because the risk that a movement examination could exaberate an existing orthopaedic condition was deemed too large.

### Movement and conformation evaluation, clinical examination- orthopaedic palpation and standing flexion tests

The clinical examinations were performed by two examiners (authors CJ and LR), both with >10 years clinical experience, during spring and autumn 2006. The examiners had no previous association with the riding schools and were not informed whether the riding schools were in the HIU or LIU group. Protocols were pre-tested at a riding school outside the study.

The horses were examined, including palpation, flexion tests, conformation evaluation and hoof inspection (see Table 1). Clinical palpation and standing flexion tests were conducted in the stable, based on a Swedish purchase examination protocol adjusted for the study. Evaluation of soreness, heat or swelling included muscles, tendons and ligaments of the neck, trunk, back and limbs (Table 1). Conformation relative to the neck, the back and the limbs was evaluated. Hoof quality and standard of hoof conformation were inspected. Standing flexion tests of the limb as a whole were performed. A standing flexion test evaluates the range of motion and pain at flexion, while not the effect on any trotting movement pattern afterwards.

**Table 1:** Distribution of findings for movement evaluation, conformation, palpation, including hoof inspection, and flexion tests in a study of 8 Swedish riding schools with 99 horses examined during 2006, the maximal numbers of horses in each category is found at the bottom row

		Total	All with remark	Ins. Cat.^1 ^(%)		Ins. Cat. and riding school (%)								Examiner (%)		Season (%)	
		No.	No.	HIU^1^	LIU^1^	HIU				LIU				CJ	LR	Spring	Autumn
Movement	Walk	99	13	6	17	11	10	0	0	35	0	6	14	17	7	20	6
evaluation	Straight trot	99	52	44	57	22	30	0	85	61	62	61	43	62	39	62	43
	Trot to the left	99	66	67	67	67	50	75	77	70	88	56	64	72	59	70	63
	Trot to the right	99	66	69	65	67	50	75	85	83	62	39	71	78	51	80	53
	Canter to the left	99	44	31	52	44	40	50	8	57	75	44	43	45	44	40	49
	Canter to the right	99	40	28	48	44	20	50	15	61	62	39	29	43	37	40	41
Conformation	Neck	95	12	9	15	0	20	0	8	10	43	17	7	13	12	9	17
	Back	99	17	14	19	0	20	25	15	26	25	0	29	24	7	24	10
	Left fore limb	95	62	69	63	67	80	100	50	70	50	61	64	61	71	63	67
	Right fore limb	95	65	77	63	78	80	100	67	60	75	61	64	65	73	63	73
	Left hind limb	95	27	37	23	33	40	0	50	45	12	17	7	31	24	35	22
	Right hind limb	95	25	31	23	33	40	0	33	45	12	17	7	28	24	30	22
Palpation	Brachiocephalicus	95	55	66	53	78	70	50	58	55	62	44	57	57	59	57	59
	Back^2^	95	53	51	58	67	30	75	50	55	62	61	57	56	56	54	57
	Saddle area	95	44	46	47	33	30	25	75	45	88	22	57	61	27	57	37
	Fore limbs	95	35	29	42	11	30	5	33	65	50	17	36	48	22	48	27
	Fore limb susp. lig.^3^	95	44	43	48	56	30	25	50	55	37	39	57	52	39	54	39
	Hind limbs	95	63	74	62	67	90	100	58	80	75	39	57	69	63	67	65
	Hind limb susp. lig.	95	21	3	33	0	0	0	8	65	37	6	21	37	2	39	8
	Hooves	95	52	49	58	56	30	25	67	65	37	67	50	57	51	61	49
Flexion tests	Fore limbs	95	46	46	50	56	40	25	50	50	50	61	36	46	51	46	51
	Hind limbs	95	61	51	72	56	20	75	67	85	75	67	57	72	54	72	57
	
	Maximal no. horses	99	99	36	63	9	10	4	13	23	8	18	14	58	41	50	49

^1 ^Ins. Cat.- insurance category, HIU- high insurance utilisation, LIU- low insurance utilisation; ^2 ^the back without the saddle area; ^3 ^suspensory ligaments

Movement at walk, trot and canter were evaluated in an outdoor or indoor arena with a sand/saw dust surface respectively, based on weather conditions. Gaits were evaluated jointly by the two examiners in the first three riding schools, and at subsequent visits only by investigator LR, due to unforeseen circumstances. Conformation, palpation and flexion tests were evaluated by investigator CJ in the first three riding schools. To set the best possible standards, before shifting to the unforeseen use of only examiner LR, criteria for conformation, palpation and flexion tests were evaluated separately and then jointly by the examiners at the fourth riding school. Thereafter, criteria were set to reflect evaluations by examiner CJ. A score of 0 was given if no observation was made or 1 if any finding was noted. All observations were registered, including minor findings without clinical relevance (i.e. not being reason for rest or treatment). The categories were thus; minor, moderate or severe.

### Data handling and analyses

Data from protocols, both dichotomised data and free text, were entered into an Excel spreadsheet (MS Excel, Microsoft Corporation, Redmond, WA 98052-6399, USA) and checked for consistency and correctness. Five indexes, summarizing 1/0 findings in each of the categories, were created; for movements (MOVE) (from "walk" to "canter on a right hand circle" in Table 1), limb conformation (CONF) (the four individual limbs under conformation, Table 1) and palpation findings for back (BACK) (back and saddle area, Table 1). The index LIMBS contained the palpatory variables brachiocephalicus, fore and hind limbs and suspensory ligaments as well as the flexion tests. A total health index included all palpatory findings (TOT-HEALTH) (variables under MOVE, CONF, BACK, fore and hind limb palpation and hooves in Table 1). The raw indexes were further adjusted for the age-distribution within the riding schools. The proportions of four age intervals (<9 years, 9 < 12 12 < 15 and ≥15 years) in the whole population (19, 30, 19, 31% respectively) were multiplied by the raw indexes to achieve adjusted indexes for each horse. The material is described using the dichotomous findings and the five unadjusted and age-adjusted indexes. Examined variables are presented by HIU/LIU-group, examiner and riding school.

The dichotomous findings were treated as dependent variables in logistic regressions, using one fixed effect and one repeated effect approach. First, riding school, with examiner nested, was forced into all models as a fixed effect. Age was tested both as linear and dummy variables. For the latter age was divided at <9 (baseline), 9 < 12, 12 < 15 and ≥15 years. When the dummy categorisation suggested that age could be treated as linear, the linear variable was used. When the age-dummies were deemed superior to the linear variable they were preferred. In the second approach, riding school was analysed as a repeated effect, with age, examiner and insurance category as fixed effects. The riding school with only four horses examined was eliminated from the logistic regressions (otherwise producing unstable models).

The individual-level indexes were analysed as dependant linear variables, based on symmetrical distributions as demonstrated by similar means and median, with examiner, breed (Swedish warmbloods vs other horses) and the three age/time-variables (Table 2) entered as independent variables (the latter tested as four-category dummies to check for linearity, similar to above and only keeping the most significant in the model). All these models included riding school as a repeated (random) effect using an exchangeable correlation. Insurance category was forced into all index models as a fixed effect. The variables with a p-value < 0.1 were entered into a primary multivariable model. Both model types were reduced manually (backwards reduction). Interactions were not tested. Variables were retained when the model p-values were below 0.15 to allow confounding variables to be kept in the models. P-values were considered significant below 0.05 and borderline from 0.05 to <0.1. In order to present the variation contributed by the riding schools, the variance estimates for riding schools were divided by the total model variation. Logistic and linear regressions were performed using PROC GENMOD and PROC MIXED respectively (SAS Institute Inc., Cary, NC, 27513, USA).

**Table 2:** Distribution of age, time at riding school, age at acquisition, crude and age-adjusted indexes (adjusted by the age distribution in the whole population) by insurance category (Ins cat- HIU/LIU high/low insurance utilisation), riding school and examiner.

	Ins cat		Riding school-Ins cat								Examiner	
	HIU	LIU	HIU				LIU				CJ	LR
Mean age (mean)	10.0	14.0	9.9	10.9	13.3	8.3	15.6	13.3	13.9	12.1	12.8	12.2
(in years) (SD)	2.9	4.6	1.8	3.1	2.6	2.3	5.5	2.7	4.2	3.7	5.0	3.7
(max)	17	25	14	16	17	12	25	17	22	19	25	22
Time at school (mean)	3.3	7.4	2.1	5.5	5.3	1.9	9.7	5.3	6.9	5.5	6.3	5.4
(in years) (SD)	3.4	4.7	2.0	5.0	2.9	1.7	4.8	4.0	4.6	3.7	4.9	4.4
Age at aquisition (mean)	6.6	6.6	7.8	5.4	8.0	6.4	5.9	8.0	6.9	6.6	6.4	6.9
(in years) (SD)	2.8	2.8	1.7	3.9	3.6	1.9	2.3	3.8	3.4	2.0	2.5	3.3
Indexes												
MOVE	2.4	3.1	2.6	2.0	2.5	2.7	3.7	3.5	2.4	2.6	3.2	2.4
CONF	2.1	1.7	2.1	2.4	2.0	2.0	2.2	1.5	1.6	1.4	1.9	1.9
BACK	1.0	1.1	1.0	0.6	1.0	1.3	1.0	1.5	0.8	1.1	1.2	0.8
LIMBS	3.1	3.5	3.2	2.8	3.3	3.3	4.6	3.9	2.7	3.2	3.8	2.9
TOT-HEALTH	7.1	7.4	7.0	6.5	7.3	7.5	8.9	8.1	6.1	6.6	7.9	6.5
Age-adjusted indexes												
MOVE	0.6	0.8	0.7	0.5	0.6	0.7	1.0	0.9	0.7	0.7	0.9	0.6
CONF	0.6	0.5	0.6	0.6	0.5	0.5	0.6	0.4	0.4	0.4	0.5	0.5
BACK	0.3	0.3	0.3	0.2	0.2	0.3	0.3	0.4	0.2	0.3	0.3	0.2
LIMBS	0.8	1.0	0.9	0.8	0.8	0.8	1.3	1.1	0.7	0.8	1.0	0.8
TOT-HEALTH	1.8	2.0	1.9	1.7	1.7	1.9	2.5	2.2	1.7	1.7	2.1	1.7

Figures are based on 92-99 horses, examined at 8 Swedish riding schools during 2006.

## Results

### The population and the findings

At the time of the examination there were 114 horses in the 8 riding schools. Nine horses of the 114 horses were not examined because they were lame or convalescent; 4 from the HIU- and 5 from the LIU-group. Six young horses at one riding school were not examined because they were undergoing training and not in normal riding school work. The remaining 99 were examined, starting with movement evaluation. Four of the 99 horses were not examined by palpation because of unforeseen time constraints. Examiner CJ examined horses at 4 riding schools (one HIU and three LIU schools).

The HIU-group included 4 riding schools and 36 horses and the LIU-group 4 riding schools and 63 horses. By riding school, the mean age of the horses varied from 8.3 to 15.6 years reflecting differences also in maximum ages between the two groups (Table 2). HIU/LIU-medians for age in general reflected the means well (data not shown). The mean height at withers in the HIU- and LIU-groups was 162 and 165 cm. The proportion of geldings was 64% and 62% in the HIU- and LIU-group. With respect to breed the proportions of Swedish warmbloods were 19% vs 56% in the HIU- and LIU-group, other Swedish or of unknown origin 22% in the HIU- and 13% in the LIU-group and imports 58% in the HIU- and 30% in the LIU-group.

The distribution of findings, overall, by insurance category, riding school, examiner and by season is seen in Table 1. The majority of the findings were judged to be of minor importance (See additional file 1: Tables 1 and 2 of specific findings in detail). In total 29 horses, from all 8 riding schools, had remarks judged of non-minor importance. Of these only four horses had remarks deemed as severe (i.e. three horses with palpatory back reactions and one with decreased forelimb joint range of motion). The number of horses with findings judged as moderate for movement was 15, for conformation 2 and for palpation 15. With respect to the moderate gait findings one horse had a remark at walk, 8 at trot (of which 6 had 2-degree lameness (0-5 scale; 0 sound and 5 non-weight bearing lameness)) and 8 at canter. Nine horses with moderate gait findings had back palpatory findings, three lower limb problems and three soreness of the brachiocephalicus muscle.

### Logistic regression analysis of findings

Age was significant for 10 findings in the logistic regression analysis (in seven riding schools, representing 91-95 horses), in at least one of the fixed or repeated effects models. As an example, if a variable is found in both Tables 3 and 4 (in Table 4 both for insurance utilisation and examiner), and for the same type of model (e.g. repeated for trot on a right hand circle), in this case the model contained all three fixed effects possible in a repeated effects model (age, insurance utilisation and examiner). Not shown in Table 3 is that for "walk" age had to be dichotomised, horses 12 years or older had a significantly higher OR (insurance utilisation, examiner and riding school all had p-values >0.15 for walk). In the fixed effect model relative to findings at walk, the OR was 21 (95% confidence interval (CI) 2-238; p = 0.001) and in the repeated effects model it was 16 (95% CI 1-209; p = 0.04), compared to younger horses. Further there was a linear increase of findings at trot on a straight line (fixed model OR 1.2 (95% CI 1.1-1.4), p = 0.006); (repeated model OR 1.1 (95% CI 1.0-1.3), p = 0.005) and palpation of fore limbs (repeated model OR 1.1 (95% CI 1.0-1.2), p < 0.0001). (For trot on a straight line there was also an examiner effect, Table 3, and for palpation of fore limbs an examiner effect, Table 4). Findings increased with age also for a majority of the significant associations, i.e. trot on a right hand circle, canter on a right hand circle, conformation left hind limb, hooves and flexion tests (Table 3). However, for conformation right fore limb the OR was lowest in the oldest age group.

**Table 3:** Odds ratios with 95% CIs^1 ^for significant (p < 0.05) and borderline (0.05 ≤ p < 0.15) age effects respective to each finding from both fixed and repeated (Rep.) effects model (seven riding schools, 91-95 horses).

			Odds ratio and 95% CI						
			
	Finding	Model	9 < 12 years		12 < 15 years		> = 15 years		p-value
Movement	Trot R^2^	Fixed	0.6	(0.2, 2.5)	2.1	(0.4, 11)	3.8	(0.8, 18)	0.06^3^
evaluation	Trot R	Rand.	1.0	(0.5, 2.1)	2.2	(0.6, 8.3)	5.3	(2.1, 14)	0.0005
	Canter L^4^	Rep.	1.8	(0.6, 5.6)	2.2	(0.4, 11)	5.4	(1.0, 2.9)	0.05^3^
	Canter R	Rep.	1.3	(0.4, 4.1)	1.6	(0.5, 5.1)	2.8	(1.1, 6.9)	0.02
Conformation	RF^5 ^limb	Fixed	0.3	(0.1, 1.7)	0.4	(0.1, 2.4)	0.1	(0.02, 0.5)	0.006
	RF limb	Rand.	0.4	(0.1, 1.2)	0.5	(0.2, 1.2)	0.1	(0.05, 0.4)	0.0007
	LH^6 ^limb	Rep.	3.4	(1.5, 7.7)	2.9	(0.5, 17)	4.6	(1.2, 17)	0.003
Palpation	Saddle area	Rep.	3.2	(1.0, 11)	1.1	(0.4, 3.2)	0.9	(0.4, 1.9)	0.05^3^
	Hind limb susp. lig.	Rep.	1.1	(0.1, 9.2)	2.4	(0.6, 9.7)	3.3	(1.0, 11)	0.05^3^
	Hooves	Fixed	1.6	(0.5, 5.2)	0.9	(0.2, 3.8)	7.5	(2.0, 29)	0.003
	Hooves	Rand.	1.6	(0.6, 3.9)	0.9	(0.3, 2.4)	7.1	(2.0, 25)	0.002
Flexion tests	F limbs	Fixed	1.1	(0.3, 3.9)	2.6	(0.6, 10)	4.4	(1.3, 16)	0.03
	F limbs	Rep.	1.1	(0.6, 2.3)	2.3	(0.8, 6.8)	3.7	(1.3, 10)	0.01
	H limbs	Rep.	0.7	(0.3, 1.4)	1.0	(0.2, 6.5)	2.5	(1.1, 5.6)	0.02

Odds ratios (1) for the baseline category (<9 years) are not shown.

^1 ^95% CI- 95% confidence intervals; ^2 ^R = right; ^3 ^borderline association; ^4 ^L = left; ^5 ^F = fore; ^6 ^H = hind

**Table 4:** Odds ratios (OR) with 95% CIs^1 ^for for significant (p < 0.05) and borderline (0.05 ≤ p <0.15) insurance utilisation and examiner effects, by finding, from repeated effects model, including seven riding schools and 91-95 horses.

	Finding	OR	95% CI	p-value
**High insurance utilisation**				
Movement	Trot on a left hand circle	1.3	(0.9, 1.7)	0.13
evaluation	Trot on a right hand circle	3.9	(2.1, 7.2)	<0.0001
	Canter on a right hand circle	0.5	(0.2, 1.2)	0.12
Conformation	Left hind limb	4.3	(1.4, 13)	0.02
Palpation	Brachiocephalicus	2.0	(1.1, 3.6)	0.03
	Hind limb suspensory ligaments	0.2	(0.1, 0.5)	0.0003

**Examinor CJ**				
Movement	Straight trot	2.3	(0.8, 6.9)	0.12
evaluation	Trot on a left hand circle	1.9	(1.7, 2.1)	<0.0001
	Trot on a right hand circle	8.0	(6.0, 11)	<0.0001
Conformation	Back	6.0	(0.9, 42)	0.07
	Right fore limb	0.8	(0.6, 1.0)	0.08
	Left hind limb	2.0	(1.3, 3.1)	0.002
Palpation	Brachiocephalicus	1.3	(0.9, 1.8)	0.12
	Saddle area	4.7	(2.8, 7.9)	<0.0001
	Fore limbs	5.0	(2.7, 9.3)	<0.0001
	Fore limb suspensory ligaments	1.8	(1.4, 2.3)	<0.0001
	Hind limb suspensory ligaments	18	(7.2, 43)	<0.0001
Flexion tests	Fore limbs	0.6	(0.4, 0.8)	0.003
	Hind limbs	2.4	(0.9, 6.3)	0.08

ORs (1) for the baseline categories (low insurance utilisation or examiner LR) are not shown.

^1 ^95% CI- 95% confidence intervals

Significant examiner effects, controlling for riding school from the repeated riding school effects models, were found eight times (Table 4). These were found for trot to the left, trot on a right hand circle, left hind limb conformation, palpation of saddle area, fore limbs, fore and hind limb suspensory ligaments and for fore limb flexion test. In only one of the eight instances, examiner CJ had fewer findings then examiner LR (OR below 1 for fore limb flexion test).

The riding school effect was evaluated in fixed effect models (with examiner nested but this has no effect on the results shown this way). Significant riding school effects were found for seven dependent variables; for trot on a straight line (p = 0.01), trot on a right hand circle (p = 0.01), canter on a left hand circle (p = 0.04), saddle area palpation (p = 0.009), fore limb palpation (p = 0.03), palpation of hind limb suspensory ligaments (<0.0001) and hind limb flexion test (p = 0.03). (Borderline significances (0.05 ≤ p < 0.1) and were found for walk, canter on a right hand circle, left hind limb conformation and hind limb palpation.)

The riding school effects were significant in absence of a significant examiner effect for three examination points; trot on a straight line, canter on a left hand circle and hind limb flexion test. A significant effect of insurance category was found four times (Table 4), controlling for riding school in a repeated effects model, i.e. for trot on a right hand circle, left hind limb conformation, and palpation of brachiocephalicus and hind limb suspensory ligaments. In two instances these coincided with the significant riding school effects as described above (trot on a right hand circle, palpation of hind limb suspensory ligaments).

### Analyses of indexes

For one of the indexes (CONF) there were no variables which showed significant differences. Examiner CJ registered more findings with respect to three of the indexes- MOVE, BACK and TOT-HEALTH. Age, time at riding school or age at acquisition was significant for these same indexes and for LIMBS. For example, while controlling for riding school for index MOVE, both examiner CJ and age, albeit not linear, were positively associated with a higher index, but insurance category was non-significant (Table 5). Breed was not significant in any model (p > 0.15). The proportion of the variation contributed by the riding schools were for CONF, MOVE and TOT-HEALTH each ≤1%, for LIMBS 10% and for BACK 4%.

**Table 5:** Results from linear regressions of examiner (comparing CJ to the baseline LR); age variables^1 ^and insurance category on the indexes, with riding school (n = 8) as a repeated (random) variable

Index Variable	Category	Estimate	SE	P-value
MOVE (n = 99)^2^	Intercept	2.39	0.42	
Examiner	CJ	0.85	0.33	0.05
Age	<9 years (BL)	0		0.01
	9 < 12 years	0.06	0.49	
	12 < 15 years	0.84	0.55	
	> = 15 years	1.64	0.51	
Insurance user	High	0.31	0.38	0.46
BACK (n = 95)	Intercept	0.69	0.16	
Examiner	CJ	0.28	0.10	0.04
Age at acquisition	< 5 years (BL)	0		0.05
	5 < 7 years	0.59	0.23	
	7 < 8 years	0.63	0.25	
	> = 8 years	0.25	0.23	
Insurance user	High	0.16	0.13	0.27
LIMBS (n = 95)	Intercept	3.34	0.47	
Time at riding school	< 2 years (BL)	0		0.03
	2 < 5 years	0.28	0.47	
	5 < 8 years	-0.64	0.49	
	> = 8 years	0.89	0.48	
Insurance user	High	-0.23	0.52	0.67
TOT-HEALTH (n = 95)	Intercept	4.49	1.05	
Examiner	CJ	1.69	0.57	0.03
Age	linear	0.25	0.07	0.00
Insurance user	High	1.18	0.66	0.13

^1 ^age, time at riding school or age at acquisition- the best form, linear or categorical, chosen during modelling and only the most significant kept; ^2 ^number of observations included

## Discussion

This is the first time that between riding schools differences in orthopaedic health status have been shown. Significant differences between riding schools were shown for seven variables in the logistic regressions. The most likely reason is differences in multifactorial management strategies that in turn influence prevention of orthopaedic injury or strategies that makes it possible to keep horses longer. Such differences between LIU and HIU riding schools appear to include variations in staff experience and/or level of training, including differences in attitude to and experience of health management (unpublished observations). One earlier study has, similar to our study (i.e. the riding school difference relative to saddle area palpation), shown differences between two riding schools regarding posture/back problems in the horses [13].

Note that the riding school and examiner effects are not fully separable. For example, examiner CJ may have visited riding schools with a different proportion of true problems in which case the CJ correction may be unnecessary and actually contribute to a conservative result or vice versa. Still, the three significant riding school effects (fixed effect model) where no examiner effect was found (repeated model) does provide the strongest evidence of a riding school effect. In the index-analyses the insurance category was insignificant when riding school was controlled for, although in the logistic regressions there were four significant associations and in three of these HIU category was a risk-factor (OR > 1).

There were substantial differences in age distribution and number of years (time) at the riding schools when comparing insurance categories and riding schools (Table 2, no statistical test performed). No difference was found for age at acquisition, supporting the hypothesis that different riding schools had strategies that prevented or increased the risk of wastage over time. These age distributions combined with the two opposite groups of insurance use, also indicates potentially differential coverage of disease data in the insurance database [14]. For example, riding schools in the LIU-group have horses with a higher median age and more years at the riding school, while the horses have been managed at a relatively low veterinary cost for several years. These horses have been managed irrespective of whether they had or did not have physical problems. However, it is likely that some have had problems that did not result in claims and thus were not registered in the insurance database, but would have been found if the same horses had the same problems at a HIU-school. Part of the differences in insurance usage may thus depend on that the LIU-schools detected horses with e.g. locomotor problems earlier and hence they were cheaper to manage, or that the HIU-schools sought expensive veterinary attention in more of these cases.

Age was statistically significant in 15 logistic regressions for 10 different outcomes (of which 10 and 7 are found in Table 3). In general findings were increased in the oldest age group, also not surprisingly, with the exception for conformation (right fore limb). In the index-regressions age (for MOVE and TOT-HEALTH), time at riding school (for LIMBS) and age at acquisition (for BACK) remained. In general, the highest age/time categories had the largest estimates, i.e. the findings were most common in the oldest age categories. Note also that two riding HIU schools did not even have horses in the oldest age category.

Whether the findings were only due to the normal degenerative effect of age or also to a rather high amount of exercise, albeit at a relatively low intensity, might be studied in longitudinal studies, comparing riding school horses to privately owned horses, both with and without high-intensity work. Meanwhile riding schools differ in how individual horses are used for lessons, including using aged horses in lighter work or culling/selling older horses (unpublished results).

Riding school, age and examiner were found to affect movement and palpation findings to a relatively large degree. With respect to conformation, for age, examiner and insurance category, only two variable were significant (right fore and left hind limb) with no significant differences among riding schools. The results are not unexpected as conformation is less likely than other findings to be influenced by management strategies or age. In addition, age was related to breed, with more old horses and in LIU-schools being Swedish warmbloods. Breed was tested in the index models but not found to be significant. However, based on this, biological reasoning and previous experience age was deemed to be of superior importance.

Because the sample was small and based on riding schools with the highest or lowest insurance utilisation generalisations to the insured or total riding school population should be done with some caution. However the sample included riding schools in both major cities and rural areas, and schools were both private and club/council-run. One may argue that this pilot study had an obvious multiple-testing problem and that some of the outcomes may be correlated. However we did want to keep the outcomes believing them to be "more specific" than when amalgamated (as we did in the index-analysis) and have used the results from these many outcomes to count the evidence instead of putting emphasis on specific outcomes. We also avoided testing too many explanatory variables in these models, even though it was deemed necessary to adjust for/investigate confounding (e.g. age, examiner or season).

The study provided more objective outside evaluation of orthopaedic status, while previous studies of incidence of injury in sport horses have often been self-reported, for example by racehorse trainers [5] or by regular veterinarians [15]. It is suggested that self-evaluation of orthopaedic status is influenced by individual experience and attitude. However, that one of the investigators had to leave the study affected the analytic strategy. In seven out of the 23 categories analysed, examiner CJ had a significant effect on the results, six times registering more findings than examiner LR. In line with previous studies this underlines the importance of controlling for between-clinician variation, e.g. large between-examiner variation has been shown when evaluating lameness [16]. The examiner effect was detected in spite of the calibration performed and the low power.

Additionally, examiner CJ (i.e. who co-examined gait with LR) was confounded with season (three riding schools visited in spring and one in autumn by CJ) and also age of the riding school horses (Table 2). In spring horses had performed 8-10 months of continuous riding school work, while in autumn they were 1-3 months after at least four weeks of annual rest at pasture. Because of this confounding the result of season has not been reported. However this also means that the examiner effect is not "true" either, being confounded by season and somewhat with age (only when not controlled for) and therefore likely exaggerated. Another problem that could influence results is horses resting due to orthopaedic problems and not examined due to safety reasons, and thus not included in the analysis. The number was 4 in the HIU and 5 in the LIU-groups, i.e. proportionately more in the HIU-group (any bias introduced likely conservative). However, this material should be regarded from the perspective that all the horses examined were currently used in riding-school work. Anecdotal evidence and preliminary analysis of a larger study on management and insurance outcome indicate that summer rest at pasture of four weeks or more has a protective effect against wastage in riding school horses (unpublished data).

Rate of insurance utilisation for orthopaedic injury was used as a means to select riding school with differences in musculoskeletal health. It was decided to a priori analyse the riding school effects in the multivariable logistic regressions, because the riding schools were selected from an historical perspective of insurance utilisation and not on the current insurance status or management. Judging subjectively at least one riding school had had major management changes between the time frame of insurance outcome analysis and the pilot study visits (data not shown). Further, though the sample size was small, the result supports the hypothesis that differences in insurance utilisation [12], are associated with differences in health status and attitude, e.g. on how insurance is used (all or some horses, early or late veterinary involvement in cases of lameness). However, the insurance category variable does not account for any variation in insurance strategies between riding schools, e.g. if only a part of the horses are insured.

The findings were further used to evaluate the status of the population and not the individuals.

As an example, among the 99 horses the most common findings for trot on a straight line were 11 findings of irregular movement on the left fore, 13 on the right fore, 10 on the left hind, 11 on the right hind, 17 of moving short and 9 of moving flat on the ground (See additional file 1: Tables 1 and 2 of specific findings in detail). As stated above most findings were of minor importance, including to many non-veterinarians hardly visible movement irregularities.

## Conclusion

This is the first time that between riding schools differences in orthopaedic health status have been shown, supporting the hypothesis that different riding school had strategies that prevented or increased the risk of wastage.

## Competing interests

The authors declare that they have no competing interests.

## Authors' contributions

CL initiated the study. CL, AE and LR designed the study. CJ and LR performed the clinical examinations. AE and CL recorded the data. AE performed the statistical analysis and drafted the manuscript. All authors read and approved the manuscript.

## Supplementary Material

Additional file 1 **Tables of specific findings for movement valuation, palpation and standing flexion tests **(Table 1) **as well as conformation **(Table 2). Click here for file

## References

[B1] Clausen M Preisinger R Kalm E Analysis of Disease in the German Warm Blood Breeding Zuchtungskunde 1990 62 167 178

[B2] Kaneene JB Ross WA Miller R The Michigan equine monitoring system. 2. Frequencies and impact of selected health problems Prev Vet Med 1997 29 277 292 10.1016/S0167-5877(96)01080-X 9234436

[B3] Penell JC Egenvall A Bonnett BN Olson P Pringle J Specific causes of morbidity among Swedish horses insured for veterinary care between 1997 and 2000 Vet Rec 2005 157 470 477 16227382 10.1136/vr.157.16.470

[B4] Mason TA Bourke JM Closure of the distal radial epiphysis and its relationship to unsoundness in two year old thoroughbreds Aust Vet J 1973 49 221 228 10.1111/j.1751-0813.1973.tb05205.x 4716444

[B5] Rossdale PD Hopes R Wingfield Digby NJ Offord K Epidemiological study of wastage among racehorses 1982 and 1983 Vet Rec 1985 116 66 69 3976145 10.1136/vr.116.3.66

[B6] Moyer W Fisher JRS Blake-Caddel L Bucked shins- effects of differing track surface surfaces and proposed training regimens Proceedings of the 37th Annual Convention of American Association of Equine Practitioners: 30 Nov 30- 2 Dec 1992; Orlando, Florida 541 547

[B7] Boston RC Nunamaker DM Gait and speed as exercise components of risk factors associated with onset of fatigue injury of the third metacarpal bone in 2-year-old thoroughbred racehorses Am J Vet Res 2000 61 602 607 10.2460/ajvr.2000.61.602 10850832

[B8] Parkin TD Clegg PD French NP Proudman CJ Riggs CM Singer ER Webbon PM Morgan KL Risk factors for fatal lateral condylar fracture of the third metacarpus/metatarsus in UK racing Equine Vet J 2005 37 192 199 10.2746/0425164054530641 15892225

[B9] Verheyen KL Newton JR Price JS Wood JL A case-control study of factors associated with pelvic and tibial stress fractures in Thoroughbred racehorses in training in the UK Prev Vet Med 2006 74 21 35 10.1016/j.prevetmed.2006.01.004 16473420

[B10] Swedish Equestrian Federation. Annual Report, Stockholm 2006

[B11] Agria Insurance Company http://www.agria.se

[B12] Egenvall A Lönnell C Roepstorff L Analysis of morbidity and mortality data in riding school horses, with special regard to locomotor problems Prev Vet Med 2009 88 298 307 10.1016/j.prevetmed.2008.10.004 19042047

[B13] Lesimple C Fureix C Menguy H Hausberger M Human direct actions may alter animal welfare, a study on horses (Equus caballus) PLoS One 2010 5 e10257 10.1371/journal.pone.0010257 20442766 PMC2860978

[B14] Mörk M Emanuelson U Lindberg A Vågsholm I Egenvall A Herd and cow characteristics affecting the odds of veterinary treatment for disease - a multilevel analysis Acta Vet Scand 2009 51 34 10.1186/1751-0147-51-34 19698112 PMC2736961

[B15] Ely ER Verheyen KLP Wood JLN Fractures and tendon injuries in National Hunt horses in training in the U.K: a pilot study Equine Vet J 2004 36 365 367 10.2746/0425164044890607 15163047

[B16] Keegan KG Wilson DA Wilson DJ Smith B Gaughan EM Pleasant RS Lillich JD Kramer J Howard RD Bacon-Miller C Davis EG May KA Cheramie HS Valentino WL van Harreveld PD Evaluation of mild lameness in horses trotting on a treadmill by clinicians and interns or residents and correlation of their assessments with kinetic gait analysis Am J Vet Res 1998 59 1370 1377 9829392

